# Metal Exposure and Associated Health Risk to Human Beings by Street Dust in a Heavily Industrialized City of Hunan Province, Central China

**DOI:** 10.3390/ijerph14030261

**Published:** 2017-03-03

**Authors:** Guangyi Sun, Zhonggen Li, Ting Liu, Ji Chen, Tingting Wu, Xinbin Feng

**Affiliations:** 1 State Key Laboratory of Environmental Geochemistry, Institute of Geochemistry, Chinese Academy of Sciences, Guiyang 550081, China; sunguangyi@mail.gyig.ac.cn (G.S.); liuting@ihb.ac.cn (T.L.); jigerchen@163.com (J.C.); wutingtingcc@163.com (T.W.); 2 University of Chinese Academy of Sciences, Beijing 100190, China; 3 Institute of Hydrobiology, Chinese Academy of Sciences, Wuhan 430072, China; 4 Guizhou Provincial Laboratory for Mountainous Environment, Guizhou Normal University, Guiyang 550001, China; 5 Key Laboratory of Karst Environment and Geohazard Prevention, Guizhou University, Guiyang 550003, China

**Keywords:** street dust, health risk, cancer, arsenic, metals

## Abstract

Fifty-five urban street dust samples were collected from Zhuzhou, an industrial city in central China and analyzed for a range of toxic elements. Potential carcinogenic and non-carcinogenic health effects on children and adults due to exposure to street dust were assessed. Concerning the two subgroups, the child cohort is confronted with considerably greater health risks than adults. According to the Hazard Quotient (HQ) method, ingestion of dust particles poses primary risk to children and adults, followed by dermal contact and inhalation for all of the metals investigated except Hg, for which inhalation of its elemental vapor constitute a slightly higher risk than ingestion. For children, Pb, As, Cd, Cr, Hg and Sb exposure were deemed as the highest contributors to non-cancer health risks, while As and Cr represent an enhanced cancer risk for children. For adults, risk indicator values for both cancer and non-cancer effects obtained were within the safety threshold. In a comparison with other locations within and outside mainland China, exposure to arsenic is prominent for the population of Zhuzhou, indicating more attention and preventive actions should been taken.

## 1. Introduction

Street dust, a special type of environmental medium with a complex composition in urban regions, could potentially cause adverse effects on exposed populations. Metal contamination character and source identification are the main concern of most previous literature on street dust [1,2,3,4,5,6]. On the basis of toxic metal contents solely, it is difficult to produce a quantitative health assessment. Although some metals are considerably enriched in street dust, both cancer and non-cancer risk normally falls below the acceptable level by the corresponding regulatory authorities [7]. Researches both from epidemiological and toxicological studies have showed that metals and metalloids can accumulate in fatty tissues, affecting the functions of organs and disrupting the nervous system or endocrinal system; or interact directly with DNA to cause mutations [8,9,10,11,12,13]. Nonetheless, in heavy impacted municipalities, there is an urgent need to assess the health risk associated with toxic metal exposure for the residents’ health. Human health risk assessment has proved to be a useful tool to pinpoint the severity of various toxic metals [14,15,16,17,18,19]. The health risk, a method combining metal pollution and human health, is developed from a qualitative or quantitative estimate of the likelihood that any of the hazards associated with the agent of concern will be realized in exposed people [20].

The objectives of the present study were to estimate health risk for children and adults due to toxic metal exposure to street dust according to Hazard Indexes (HI) and cancer risk assessment in Zhuzhou, a heavily industrialized city in China, and put the result into a broader perspective of similar studies in China and elsewhere.

## 2. Materials and Methods

### 2.1. Study Area

**Figure 1:**
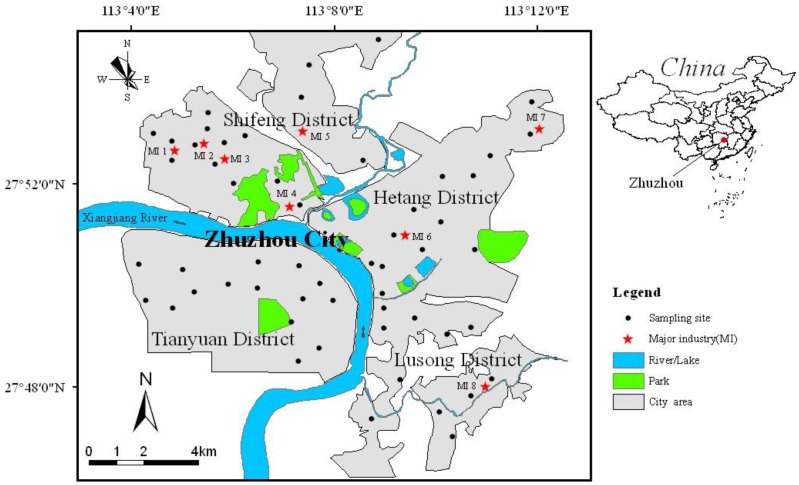
Sketch map showing the study area with sampling locations of street dust and the major industries in Zhuzhou city.

Zhuzhou, the second largest city of Hunan Province in central China, is located in the middle reaches of the Xiang River watershed (Figure 1) and about 50 km south of Changsha, the provincial capital. The city developed rapidly from a town in the 1950s to a large city, with a population of 1.002 million and a built-up area of 105 km^2^ by the end of 2009. Zhuzhou has a typical north subtropical monsoon climate, with prevailing NNW winds, except during summer (June–August) with SE winds. Its annual average temperature is 17.6 °C with plenty of precipitation, mainly during March to August, with an annual rainfall of 1409 mm. The topography mostly consists of alluvial plains and gentle hills at an elevation of 50–200 m above sea level. Acidic ferro soils of krasnozem type are here predominant as elsewhere in southern China [21].

Zhuzhou is a major base of heavy industries in Hunan Province. Zhuzhou Electrical Railway Engine Corporation, Zhuzhou Hard Alloy Group, China Southern Power Equipment Corporation and Zhuzhou Smelting Group are all backbone enterprises of the city, predominantly established during the era of China′s first and second “Five-year Plan” (1953–1962). The city’s industrial output is characterized by production foremost in the categories of metallurgy, machinery, chemistry and building materials [21].

### 2.2. Data Sources

The street dust samples were collected in winter of 2010. A total of 55 samples were obtained across the city, as shown in Figure 1. The levels, spatial distributions and possible sources of heavy metals (Ag, As, Cd, Co, Cr, Cu, Hg, Mo, Ni, Pb, Sb, Zn) in street dust were reported by our previous publication (Li et al. [21]). The data showed that the Zn/Pb smelting activities located in the northwest part of city are the main source of Ag, As, Cd, Cu, Hg Pb, Sb and Zn, while a hard alloy plant in the central city responsible for the Mo enrichment, nevertheless Co, Cr and Ni are mainly ascribed to the natural sources.

### 2.3. Risk Assessment Method

Human exposure to trace elements in street dust can induce health risks via three main paths: (a) direct ingestion of substrate particles (*D_ing_*); (b) inhalation of suspended particles through mouth and nose (*D_inh_*); and (c) dermal absorption of potentially harmful elements in particles adhered to exposed skin (*D_dermal_*). The dose received through each of the three pathways was calculated using Equations (1)–(3), which were adopted from U.S. Environmental Protection Agency [22,23] and Zheng et al. [15,16]. For Hg, exposure via inhalation of its elemental vapor is viable following Equation (4). For carcinogens, lifetime average daily dose (LADD) is used for assessment of cancer risk. LADD is calculated as a weighted average for each exposure route as given in Equation (5):

(1) $$D_{ing}=C\times \frac{IngR\times EF\times ED}{BW\times AT}\times 10^{-6}$$

(2) $$D_{inh}=C\times \frac{InhR\times EF\times ED}{PEF\times BW\times AT}$$

(3) $$D_{dermal}=C\times \frac{SL\times SA\times ABS\times EF\times ED}{BW\times AT}\times 10^{-6}$$

(4) $$D_{vapour}=C\times \frac{InhR\times EF\times ED}{VF\times BW\times AT}$$

(5) $$LADD=C\times \frac{CR\times EF\times ED}{PEF\times BW\times AT}$$

The formulae acronyms used in Equations (1)–(5) are explained in Table 1. The reference values used in the analysis were retrieved from U.S. EPA [24], Van den Berg [19], Naturvårdsverket [25] and De Miguel [24]. Site specific biometric factors were assessed from literature recommendations [19,24].

C (exposure-point concentration, mg·kg^−1^) in Equations (1)–(5), combined with the values of exposure factors shown above, are considered to yield an estimate of the “reasonable maximum exposure” [22], which is the upper limit of the 95% confidence interval for mean (UCL, upper confidence limit) [23]. Considering that the concentration of each trace element investigated followed log-normal distribution among samples, 95% UCL was calculated using the statistical software SPSS 15.0 for Windows (SPSS Inc., Chicago, IL, USA).

The potential carcinogenic and non-carcinogenic risks for human being were calculated using Equations (6) and (7) [26].

Carcinogenic Risk = *LADD × SF* (6)

Hazard quotient (HQ) = *D_i_/RfD* (7)

For carcinogens, the carcinogenic risk is multiplied by the corresponding slope factor (SF) to produce a level. The acceptable or tolerable risk for regulatory purposes is in the range of 1 × 10^−6^–1 × 10^−4^ [19]. RfD is the reference dose (mg·kg^−1^·day^−1^) and D_i_ is the daily exposure amounts of the selected metal (mg·kg^−1^·day^−1^). Hazard Quotient (HQ) for non-carcinogenic effects was applied to each exposure pathway in the analysis. The approach assumes that simultaneous sub-threshold exposures to toxic metals could result in adverse health effects and the magnitude of the adverse effect will be proportional to the sum of the ratios of the sub-threshold exposures to acceptable exposures [22]. Hazard index (HI) is equal to the sum of HQ. If the value of HI is less than one, it is assumed that there is no significant risk of non-carcinogenic effects. If HI exceeds one, then there is a chance that non-carcinogenic effects occur, with a probability which tends to increase as the value of HI increases [24]. For a comparison, relative toxicity values (RfD and SF) from this study and from the literature are summarized in Table 2.

## 3. Results and Discussion

### 3.1. Trace Elements in the Street Dust

Descriptive statistical results of trace element concentrations investigated in Zhuzhou city, as well as their background value in surface soils of Hunan province [30], are summarized in Table 3. In particular, the average concentration of Zn and Pb is enhanced at 2379 mg·kg^−1^ and 956 mg·kg^−1^, respectively. The arithmetic mean concentration for elements followed in the order of Zn > Pb > Cu >Cr > As >Cd > Ni > Sb > Co > Mo > Ag > Hg.

The concentration distribution in samples for all of the studied elements display a positive skewness (>1) and can be approximated as a log-normal one. Furthermore, enrichment factors (EFs) for all the elements except for Co are greater than unity signifying anthropogenic influences. Among the elements, the magnitude of EF peaking for Cd (81.75) but is also considerably high (>10) for some adjacent elements in the periodical table, namely Zn and Ag.

### 3.2. Non-Carcinogenic Risk of Metals

For non-carcinogenic health effects among children and adults, the calculated overall risk and the corresponding contribution for each exposure pathway are summarized in Table 4. In the case of children, the rank for exposure risk among the three routes was for all elements ingestion > dermal contact > inhalation. Hg is an exception, for which inhalation of Hg vapor is the primary cause of exposure and not by particulate matter [13]. This result also applies to adults. Similar results were obtained by Ferreira-Baptista and DeMiguel [14], and Zheng et al. [21,22].

In general, children exposure non-cancer risks of toxic metals were nearly one order of magnitude higher than the values of adult. So, children are confronted by greater harmful health risks due to the street dust than other population groups. This is similar to other reports [7].

HIs for metals to children and adult decrease in the order of Pb > As > Cd > Sb > Cr > Hg > Zn > Cu > Ni > Mo > Co > Ag and Pb > As > Hg > Cd > Sb > Cr > Zn > Cu > Ni > Mo > Co > Ag, respectively, The above showed that Pb/Zn smelting may be the most important factor raising risk to human health, due to Pb mainly related to Pb/Zn smelting. Except for Pb and As, HIs for other metals due to street dust exposure in this study are lower than the safety limit of unity, indicating less non-cancer risks from these elements for children and adult.

Concerning the two population groups, they both experience potential health risk by Pb and As exposure from street dust. For children, the Hazard Indexes for Pb and As exceed the safe level (both greater than 3, Table 4). There has a great possibility to pose potential adverse health effects to human beings that non-carcinogenic effects may occur, with a probability which tends to increase as the value of HI increases [15,16,24]. According to De Miguel et al. [31], adverse health effects may occur at HI values >0.1 level in the child cohort. Consequently, Cd, Cr, Hg and Sb, that with HIs greater than 0.1 for the child, exposure to the street dust in Zhuzhou cannot be overlooked, and its ecological and health implications need investigated in detail in the further.

### 3.3. Risks Associated with the Carcinogenic Elements As, Cd, Cr, Co and Ni

The overall carcinogenic risks owing to exposure to As, Cd, Co, Cr and Ni in street dust are shown in Table 5. The carcinogenic risk levels of Cd, Co and Ni exposure for both cohorts are within the same low order of magnitude (10^−8^~10^−9^), which means that they pose a negligible risk [32,33].

In terms of children, the calculated value for As (1.04 × 10^−6^) and Cr (1.07 × 10^−6^) in street dust falls within the range of threshold values (10^−4^–10^−6^) which is an internationally accepted precautionary risk criterion [32,33]. In contrast, adult exposure carcinogenic risk for these elements falls below this threshold (1.29 × 10^−7^ and 6.05 × 10^−7^ for As and Cr, respectively). As in street dust is introduced by the nonferrous smelting activities and Cr is naturally enriched in the local soil of Zhuzhou [21]. As a carcinogen, arsenic can cause lung and skin cancer [34] and Cr can trigger lung cancer and stomach cancer [34] if an excessive buildup of both elements occurs. In view of the above, the risk of lung cancer disease increased by one times for the two subpopulations. Hence, further research should be undertaken to reveal the synergistic influence from Cr and As on human health.

### 3.4. Comparison of HI and Cancer Risk with Other Researches

The HI and cancer risk assessment results in street dust of Zhuzhou were compared with results from other cities around the world, as shown in Table 6 and Table 7. For children, there is only one additional study (Huludao, Liaoning Province) that reported risk values of Cd and Hg exceeding 0.1. Huludao city once hosted the Asia’s biggest zinc manufacturer. In addition, a study in Dongying city with large petrochemical industries reveal a HI larger than 0.1 for Cr [35], which was likewise found in this study. Similar discovery were obtained in municipal areas of Shanghai by Shi et al. [7] in a study of health risk of potentially metals in road dust.

Furthermore, hazard indexes of As and Pb for children exposure with value higher 10^−1^ appeared in several studies (see Table 6), so, these two elements could trigger neurological and developmental disorders [14]. As the same time, arsenic was the only element that associated with carcinogenic risk exceeding 10^−6^ in the reviewed studies. As and Pb appear to have a widespread impact on children health in polluted cities all over the world.

**Figure 2:**
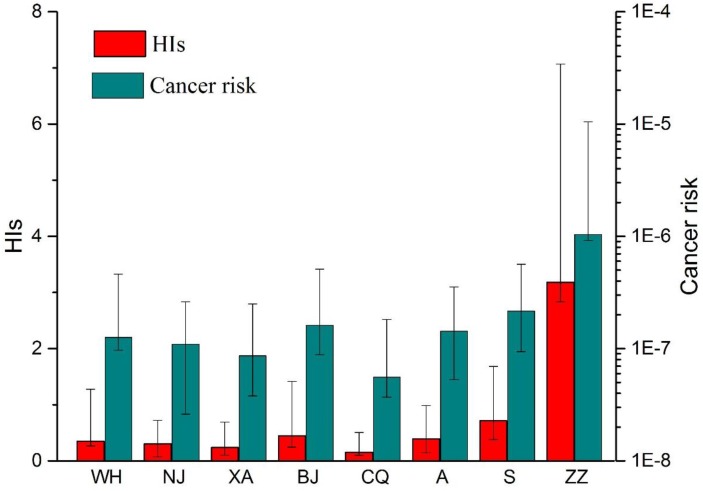
A comparison of HIs and cancer risk of arsenic to children in street dust of Wuhu (WH), Nanjing (NJ), Xi’an (XA), Baoji (BJ), Chongqing (CQ), Avilés (A), Singapore (S) and this study (ZZ).

Since arsenic is outstanding both for cancer and non-cancer risk for many researches. Here we take arsenic as an example, compared the results obtained by this study and other researches. The HI and cancer risk of As for children are calculated according to the date from other literatures [33,36,37,38,39]. The HI means of these cities are less than 0.1 (Figure 2) and the cancer risk means are in 10^−7^ to 10^−8^ level (Figure 2). The maximum of HI in Wuhu (China) and Singapore are 9.29 × 10^−1^, 9.66 × 10^−1^, respectively, which are very close to one. While the data indicated that the HI and cancer risk of As in Zhuzhou are much serious than other cities (HI > 3, and cancer risk > 10^−6^), indicating a more worrying polluted status.

Meanwhile, for adults, HIs for Ag, As, Cd, Co, Cr, Hg, Sb and Zn in these cities (Zhuzhou, Huludao and Dongying are lower than 1, suggesting that there is little adverse health risk due to street dust. From the aforementioned statements, it could be concluded that As and Pb contamination has become a serious environmental problem in a category of cities and further research should be undertaken to explore the particular influence of As on children health.

## 4. Conclusions

This study elucidates that children’s health is specifically at risk when exposed to street dust enriched in toxic metals and metalloids such as Pb and As, as the case study in Zhuzhou city of Hunan Province, China, showed. Hazard quotient calculations indicate that ingestion of dust particles pose the highest risk to children and adults in Zhuzhou, followed by dermal contact and subsequently inhalation for all the metals included except Hg, for which inhalation of its vapor presents a slightly higher risk than ingestion. For adults, risk values of both carcinogenic and non-carcinogenic health effects in this study were in the receivable range. For children, Pb, As, Cd, Cr, Hg and Sb are regarded as the most possible contributor to non-carcinogenic health risks, while As and Cr increase the risk for causing cancer. Compared the HI and cancer risk of arsenic for children in this study to others, we found arsenic was regarded as the most possible element to cancer and non-cancer risks for children in those cities (Huludao, Dongying, Luanda, Madrid). Hence, further study should pay more attention to the effects of As in dust on children’s health.

## Figures and Tables

**Table 1:** Description of parameters and the default values used in does and health risk assessment.

Symbol	Meanings	Default Value	Data Source, Year [Reference]
C	Value of elements in street dust	95% UCL	This study
*IngR*	Ingestion rate	200 mg·day^−1^ (child); 100 mg·day^−1^(adult)	U.S. EPA, 2001 [24]
*InhR*	Inhalation rate	7.6 m^3^·day^−1^ (child); 20 m^3^·day^−1^ (adult)	Van den Berg, 1995 [27]
*EF*	Exposure frequency (site specific)	180 day·year^−1^	Ferreira-Baptista and De Miguel, 2005 [14]
*ED*	Exposure duration	6 year (child); 24 year (adult)	U.S. EPA, 1996, 2001 [23,24]
*SA*	Skin area exposed	1150 cm^2^(child); 2145 cm^2^(adult)	Wang et al. 2008 [28]
*SL*	Skin adherence factor	0.2 mg·cm^−2^·day^−1^(child); 0.07 mg·cm^−2^·day^−1^(adult)	U.S. EPA, 1996, 2001 [23,24]
*ABS*	Dermal absorption factor	0.03 (for As); 0.001 (for other elements)	U.S. DOE, 2004 [20]
*PEF*	Particle emission factor	1.36 × 10^9^ m^3^·kg^−1^	U.S. EPA, 2001 [24]
*VF*	Volatility factor	32,675.6 m^3^·kg^−1^ (elemental mercury vapor)	U.S. EPA, 2001 [24]
*BW*	Body weight	15.4 kg (child); 56 kg (adult)	U.S. EPA, 1996, 2001;Wang et al. 2005 [23,24,29]
*AT*	Average time	*ED* × 365 (for non-carcinogens); *ED* × 365 (for carcinogens)	U.S. EPA, 1989 [22]
*CR*	Contact (absorption) rate	Ingestion: [*CR* = *IngR*]; Inhalation: [*CR* = *InhR*]; Dermal: [*CR* = *SA* × SL × ABS]	U.S. EPA, 1996, 2001 [23,24]

**Table 2:** Relative toxicity values used in this study [14,15,16,23].

	Ag	As	Cd	Co	Cr	Cu	Hg	Mo	Ni	Pb	Sb	Zn
Oral RfD	5.00 × 10^−3^	3.00 × 10^−4^	1.00 × 10^−3^	2.00 × 10^−2^	3.00 × 10^−3^	4.00 × 10^−2^	3.00 × 10^−4^	5.00 × 10^−3^	2.00 × 10^−2^	3.50 × 10^−3^	4.00 × 10^−4^	3.00 × 10^−1^
D × 10 rmal. RfD	9.00 × 10^−4^	1.23 × 10^−4^	1.00 × 10^−5^	1.60 × 10^−2^	6.00 × 10^−5^	1.20 × 10^−2^	2.10 × 10^−5^	1.90 × 10^−3^	5.40 × 10^−3^	5.25 × 10^−4^	8.00 × 10^−6^	6.00 × 10^−2^
Inhal. RfD	5.00 × 10^−3^	3.00 × 10^−4^	1.00 × 10^−3^	5.71 × 10^−6^	2.86 × 10^−5^	4.02 × 10^−2^	2.90 × 10^−4^	4.95 × 10^−3^	2.06 × 10^−2^	3.52 × 10^−3^	4.00 × 10^−4^	3.00 × 10^−1^
Vapour RfD							8.57 × 10^−5^					
Oral SF		1.50 × 10										
D × 10 rmal SF		3.66 × 10										
Inhal. SF		1.51 × 10	6.30 × 10	9.80 × 10	4.20 × 10^1^				8.40 × 10^−1^			

**Table 3:** Trace elements concentrations in street dust collected form Zhuzhou city [21] (N = 55, mg·kg^−1^).

Element	Median	Mean	SD	Min	Max	95% UCL	Skewness	Reference Value	EF
Ag	1.17	2.49	4.56	0.46	28.7	3.72	4.49	0.108	10.83
As	41.8	87.8	182.9	15.4	1194.1	137.2	5.05	15.7	2.7
Cd	10.3	41.4	117.3	2.2	691	73.1	4.71	0.126	81.75
Co	13	15	11	8	84	18	4.85	14.6	0.86
Cr	115	124.6	54.1	59.5	302	139	1.78	71.4	1.61
Cu	97.6	139	148.3	39.2	1020	179	4.44	27.3	3.58
Hg	0.21	0.92	2.7	0.08	14.6	1.65	4.69	0.116	1.77
Mo	3.2	6.4	12.4	1.3	90.1	9.7	6.02	1.4	2.25
Ni	35	40	16	20	105	45	1.95	31.9	1.1
Pb	254	956	2815	96	17,578	1717	5.03	29.7	8.54
Sb	9.8	15.8	21.7	3.7	115.4	21.7	3.81	1.87	5.24
Zn	1140	2379	5145	317	35,400	3770	5.46	94.4	12.08

SD: Std. Deviation; Min: Minimum; Max: Maximum; Reference value in the soil of Hunan province, (CNEMC, 1990) [30]; EF, enrichment factor = median/reference value.

**Table 4:** Daily exposure amounts and hazard quotient of metals in street dust to adult and child through three routes.

mg·kg^−1^·day^−1^	Ag	As	Cd	Co	Cr	Cu	Hg	Mo	Ni	Pb	Sb	Zn
Adult												
Ding	3.28 × 10^−6^	1.21 × 10^−4^	6.44 × 10^−5^	1.59 × 10^−5^	1.22 × 10^−4^	1.58 × 10^−4^	1.45 × 10^−6^	8.54 × 10^−6^	3.96 × 10^−5^	1.51 × 10^−3^	1.91 × 10^−5^	3.32 × 10^−3^
Dd × 10 rmal	4.92 × 10^−9^	5.44 × 10^−6^	9.67 × 10^−8^	2.38 × 10^−8^	1.84 × 10^−7^	2.37 × 10^−7^	2.18 × 10^−9^	1.28 × 10^−8^	5.95 × 10^−8^	2.27 × 10^−6^	2.87 × 10^−8^	4.98 × 10^−6^
Dinh	4.82 × 10^−10^	1.78 × 10^−8^	9.47 × 10^−9^	2.33 × 10^−9^	1.80 × 10^−8^	2.32 × 10^−8^	2.14 × 10^−10^	1.26 × 10^−9^	5.83 × 10^−9^	2.22 × 10^−7^	2.81 × 10^−9^	4.88 × 10^−7^
Dvapour							8.89 × 10^−6^					
HQing	6.55 × 10^−4^	4.03 × 10^−1^	6.44 × 10^−2^	7.93 × 10^−4^	4.08 × 10^−2^	3.94 × 10^−3^	4.84 × 10^−3^	1.71 × 10^−3^	1.98 × 10^−3^	4.32 × 10^−1^	4.78 × 10^−2^	1.11 × 10^−2^
HQd × 10 rmal	5.47 × 10^−6^	4.42 × 10^−2^	9.67 × 10^−3^	1.49 × 10^−6^	3.06 × 10^−3^	1.97 × 10^−5^	1.04 × 10^−4^	6.75 × 10^−6^	1.10 × 10^−5^	4.32 × 10^−3^	3.59 × 10^−3^	8.31 × 10^−5^
HQinh	9.64 × 10^−8^	5.92 × 10^−5^	9.47 × 10^−6^	4.08 × 10^−4^	6.29 × 10^−4^	5.77 × 10^−7^	7.37 × 10^−7^	2.54 × 10^−7^	2.83 × 10^−7^	6.32 × 10^−5^	7.03 × 10^−6^	1.63 × 10^−6^
HQvaour							1.04 × 10^−1^					
HI = ∑HQ	6.61 × 10^−4^	4.47 × 10^−1^	7.40 × 10^−2^	1.20 × 10^−3^	4.45 × 10^−2^	3.96 × 10^−3^	1.09 × 10^−1^	1.72 × 10^−3^	1.99 × 10^−3^	4.36 × 10^−1^	5.14 × 10^−2^	1.12 × 10^−2^
Child												
Ding	2.38 × 10^−5^	8.79 × 10^−4^	4.68 × 10^−4^	1.15 × 10^−4^	8.90 × 10^−4^	1.15 × 10^−3^	1.06 × 10^−5^	6.21 × 10^−5^	2.88 × 10^−4^	1.10 × 10^−2^	1.39 × 10^−4^	2.41 × 10^−2^
Dd × 10 rmal	2.74 × 10^−8^	3.03 × 10^−5^	5.38 × 10^−7^	1.33 × 10^−7^	1.02 × 10^−6^	1.32 × 10^−6^	1.22 × 10^−8^	7.14 × 10^−8^	3.31 × 10^−7^	1.26 × 10^−5^	1.60 × 10^−7^	2.78 × 10^−5^
Dinh	6.66 × 10^−10^	2.46 × 10^−8^	1.31 × 10^−8^	3.22 × 10^−9^	2.49 × 10^−8^	3.20 × 10^−8^	2.95 × 10^−10^	1.74 × 10^−9^	8.05 × 10^−9^	3.07 × 10^−7^	3.88 × 10^−9^	6.75 × 10^−7^
Dvapour							1.23 × 10^−5^					
HQing	4.76 × 10^−3^	2.93 × 10	4.68 × 10^−1^	5.76 × 10^−3^	2.97 × 10^−1^	2.87 × 10^−2^	3.52 × 10^−2^	1.24 × 10^−2^	1.44 × 10^−2^	3.14 × 10	3.47 × 10^−1^	8.05 × 10^−2^
HQd × 10 rmal	3.04 × 10^−5^	2.46 × 10^−1^	5.38 × 10^−2^	8.29 × 10^−6^	1.71 × 10^−2^	1.10 × 10^−4^	5.79 × 10^−4^	3.76 × 10^−5^	6.14 × 10^−5^	2.41 × 10^−2^	2.00 × 10^−2^	4.63 × 10^−4^
HQinh	1.33 × 10^−7^	8.18 × 10^−5^	1.31 × 10^−5^	5.64 × 10^−4^	8.70 × 10^−4^	7.97 × 10^−7^	1.02 × 10^−6^	3.51 × 10^−7^	3.91 × 10^−7^	8.73 × 10^−5^	9.71 × 10^−6^	2.25 × 10^−6^
HQvaour							1.43 × 10^−1^					
HI = ∑HQ	4.80 × 10^−3^	3.18 × 10	5.22 × 10^−1^	6.34 × 10^−3^	3.15 × 10^−1^	2.88 × 10^−2^	1.79 × 10^−1^	1.25 × 10^−2^	1.45 × 10^−2^	3.17 × 10	3.67 × 10^−1^	8.09 × 10^−2^

**Table 5:** Daily exposure amounts and Cancer Risk of metals in street dust to adult and child through three routes.

mg·kg^−1^·day^−1^	As	Cd	Co	Cr	Ni
Adult					
LADD-Oral	8.88 × 10^−8^				
LADD-Dermal	4.00 × 10^−9^				
LADD-Inhal.	1.78 × 10^−8^	9.47 × 10^−9^	2.33 × 10^−9^	1.80 × 10^−8^	5.83 × 10^−9^
Risk-Oral	1.33 × 10^−7^				
Risk-Dermal	1.46 × 10^−8^				
Risk-Inhal	2.68 × 10^−8^	5.96 × 10^−8^	2.28 × 10^−8^	7.56 × 10^−7^	4.90 × 10^−9^
Risk-Total	1.75 × 10^−7^	5.96 × 10^−8^	2.28 × 10^−8^	7.56 × 10^−7^	4.90 × 10^−9^
Child					
LADD-Oral	6.46 × 10^−7^				
LADD-Dermal	2.23 × 10^−8^				
LADD-Inhal.	2.46 × 10^−8^	1.31 × 10^−8^	3.22 × 10^−9^	2.49 × 10^−8^	8.05 × 10^−9^
Risk-Oral	9.69 × 10^−7^				
Risk-Dermal	8.16 × 10^−8^				
Risk-Inhal	3.71 × 10^−8^	8.24 × 10^−8^	3.16 × 10^−8^	1.04 × 10^−6^	6.76 × 10^−9^
Risk-Total	1.09 × 10^−6^	8.24 × 10^−8^	3.16 × 10^−8^	1.04 × 10^−6^	6.76 × 10^−9^

**Table 6:** Comparisons of hazard quotient for trace metals in urban dust of different cities.

City	Factor	Ag	As	Cd	Co	Cr	Cu	Hg	Mo	Ni	Pb	Sb	Zn	Literature
Adult														
Zhuzhou, China	Industrial pollution	6.61 × 10^−4^	4.47 × 10^−1^	7.40 × 10^−2^	1.20 × 10^−3^	4.45 × 10^−2^	3.96 × 10^−3^	1.09 × 10^−1^	1.72 × 10^−3^	1.99 × 10^−3^	4.36 × 10^−1^	5.14 × 10^−2^	1.12 × 10^−2^	This study
Huludao, China	Zinc smelting			2.58 × 10^−1^			5.71 × 10^−3^	8.79 × 10^−2^			1.44 × 10^−1^		1.40 × 10^−2^	Zheng et al. 2010 [15]
Dongying, China	Oil industry		6.73 × 10^−2^	2.46 × 10^−3^	7.46 × 10^−4^	2.24 × 10^−2^	1.85 × 10^−3^			1.86 × 10^−3^	4.21 × 10^−2^		2.23 × 10^−3^	Kong et al. 2011 [17]
Child														
Zhuzhou, China	Industrial pollution	4.80 × 10^−3^	3.18 × 10	5.22 × 10^−1^	6.34 × 10^−3^	3.15 × 10^−1^	2.88 × 10^−2^	1.79 × 10^−1^	1.25 × 10^−2^	1.45 × 10^−2^	3.17 × 10	3.67 × 10^−1^	8.09 × 10^−2^	This study
Huludao, China	Zinc smelting			6.16 × 10^−1^			4.75 × 10^−2^	1.82 × 10^−1^			1.08 × 10		1.11 × 10^−1^	Zheng et al. 2010 [15]
Dongying, China	Oil industry		5.86 × 10^−1^	2.10 × 10^−2^	5.04 × 10^−3^	1.96 × 10^−1^	1.73 × 10^−2^				3.90 × 10^−1^		2.07 × 10^−2^	Kong et al. 2011 [17]
Luanda, Angola	Industrial pollution	8.42 × 10^-4^	1.36 × 10^−1^	1.01 × 10^−2^	1.11 × 10^−3^	6.63 × 10^−2^	7.30 × 10^−3^	1.61 × 10^−2^	2.81 × 10^−3^	3.55 × 10^−3^	7.23 × 10^−1^	6.58 × 10^−2^	7.40 × 10^−3^	Ferreira−Baptista and De Miguel, 2005 [14]
Madrid, Spain	Industrial pollution	1.67 × 10^−4^	1.09 × 10^−1^	1.09 × 10^−3^		2.72 × 10^−2^	1.41 × 10^−3^	1.71 × 10^−2^	6.00 × 10^−4^	9.61 × 10^−4^	3.11 × 10^−2^	1.06 × 10^−2^	7.10 × 10^−4^	De Miguel et al. 2007 [31]

**Table 7:** Comparisons of cancer risk for child in urban dust of different cities.

City	Factor	As	Cd	Co	Cr	Ni	Literature
Zhuzhou, China	Industrial pollution	1.04 × 10^−6^	8.46 × 10^−8^	3.24 × 10^−8^	1.07 × 10^−6^	6.94 × 10^−9^	This study
Huludao, China	Zinc smelting				2.36 × 10^−8^		Zheng et al. 2010 [15]
Dongying, China	Oil industry	7.21 × 10^−7^	8.06 × 10^−10^	4.35 × 10^−8^		2.94 × 10^−8^	Kong et al. 2011 [17]
Luanda, Angola	Industrial pollution	7.69 × 10^−6^	3.89 × 10^−10^	1.53 × 10^−9^	5.70 × 10^−8^	4.61 × 10^−10^	Ferreira-Baptista and De Miguel, 2005 [14]
Madrid, Spain	Industrial pollution	4.19 × 10^−6^	2.62 × 10^−11^		1.73 × 10^−8^	1.37 × 10^−9^	De Miguel et al. 2007 [31]
